# Reversible DNA micro-patterning using the fluorous effect†
†Electronic supplementary information (ESI) available: Experimental procedures and further data. See DOI: 10.1039/c7cc00288b



**DOI:** 10.1039/c7cc00288b

**Published:** 2017-02-23

**Authors:** Gabriella E. Flynn, Jamie M. Withers, Gerard Macias, Justin R. Sperling, Sarah L. Henry, Jonathan M. Cooper, Glenn A. Burley, Alasdair W. Clark

**Affiliations:** a Biomedical Engineering Research Division , School of Engineering , University of Glasgow , Rankine Building , Oakfield Avenue , Glasgow , UK . Email: Alasdair.clark@glasgow.ac.uk; b WestCHEM & Department of Pure & Applied Chemistry , University of Strathclyde , 295 Cathedral Street , Glasgow , G1 1XL , UK . Email: glenn.burley@strath.ac.uk

## Abstract

We describe a new method for the immobilisation of DNA into defined patterns with sub-micron resolution, using the fluorous effect. The method is fully reversible *via* a simple solvent wash, allowing the patterning, regeneration and re-patterning of surfaces with no degradation in binding efficiency following multiple removal/attachment cycles of different DNA sequences.

## Abstract 2

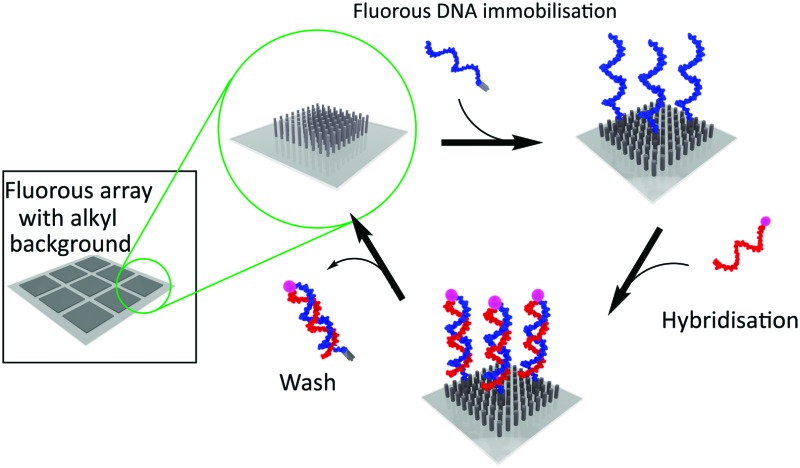
We described the use of the fluorous effect as a simple and reversible immobilisation technique for DNA.

Of central focus to many chemical studies is molecular surface patterning, due to the potential impact of this research in scientific fields ranging from biosensing and diagnostics to computing.^1^ Of particular interest is the patterning of biomolecules, more specifically DNA. This is due to its propensity for specific molecular recognition and its ability to self-assemble.^2^ As such, its unique structural and chemical properties have seen it employed in an increasingly diverse range of applications. From biological screening to materials assembly, the integration of micro- and nano-patterned DNA with solid supports is being used to develop novel devices that bridge the gap between organic and inorganic engineering.^3–5^ As a result, efficient and flexible surface immobilization chemistries are paramount to the future development of DNA-based technologies. These chemistries must adhere to a number of stringent requirements: accessibility of the surface-bound DNA strands; intact functionality of the DNA base-pairing mechanism; high density of attachment; low non-specific binding; and high array stability. However, many immobilisation strategies fail to meet these requirements.^6^ Additionally, they tend to be static in nature, providing no opportunity to modify the composition of the DNA pattern after initial immobilisation.^7–9^ This restricts the potential applications of these surfaces, particularly in biosensing and diagnostics, where such devices are limited to a single use.^10^ Therefore, the development of chemistries that enable reversible and rewritable patterning would provide a route towards more versatile systems and devices. To that end, reversible DNA patterning has been demonstrated as a means to create complex micro-arrays for reusable screening applications.^11,12^ Furthermore, dynamic DNA patterns could provide a route towards a surface-based implementation of the transformable nanoparticle systems, which has been demonstrated recently in solution, for applications in reconfigurable nanophotonics.^13^

Currently, reusable DNA microarray chips typically rely on a stripping mechanism to refresh the surfaces between each use.^14^ This denatures the double stranded DNA leaving only the covalently bound single strand on the surface, which is then available to bind once more to the target. This process has many benefits, including reduced cost and higher consistency between experiments.^15^ Typically, high temperature detergents are required to strip the surface, which are potentially detrimental to the integrity of the chip. Furthermore, this technique only allows for the same probes to be screened multiple times.^14^ As a result, investigations concerning the development of new re-writable DNA platforms have become more widespread, and have concentrated on the use of disulphide bonds as reversible anchors for DNA immobilisation, as these covalent linkages are capable of reversible cleavage.^16,17^ Although these methods offer strong binding, they require a large excess of reagent to achieve an efficient regeneration, and are often limited by surface degradation after multiple cycles (due to, for example, thiol oxidation on surfaces).^17^ In contrast, fluorous surfaces are chemically inert, and the non-covalent nature of the “fluorous effect” allows for the complete removal of surface-bound biomolecules using simple solvent washes.^18^ In this paper, we show for the first time the implementation of the fluorous effect for the reversible immobilisation of DNA micro-patterns on solid supports.

The fluorous effect refers to the observation that highly fluorinated or perfluorinated compounds have a tendency to exclude themselves from both aqueous and organic phases.^19^ The resulting effect of this phenomenon is the product of reduced London dispersion forces between per-fluorinated compounds as a consequence of the extremely low polarizability of the C–F bond. Molecular tagging with fluorous “ponytails” has the effect of drawing compounds into fluorous layers – a feature that has been widely exploited in synthetic chemistry to facilitate product purification.^19^ ”Fluorous affinity” tags have also been used as an effective means of immobilising carbohydrates and peptides on surfaces, and in screening protein-small molecule interactions.^18–26^ Existing examples employ mechanical means to introduce samples, relying upon printing, stamping, or direct spotting of fluorous-tagged compounds in order to create defined, two-dimensional microarrays of molecular information.^21–26^ The major advantages of this technology are the low sample volumes required and the ability for multiplexing, whereby multiple targets can be screened for on the same chip. However, a new printing/stamping step is required each time the surface is to be re-used. This is also true of the fabrication of many DNA microarray chips.^27^ As a consequence, these techniques are often slow, are difficult to scale-up, and may rely on expensive printing devices.

In this manuscript we demonstrate that (i) short fluorous tags immobilise water-soluble oligodeoxyribonucleotides (ODNs) from aqueous solutions onto lithographically-defined, micro-patterned fluorous surfaces without the need to direct sample spotting; (ii) ODNs immobilised by this method remain available to hybridise with a complementary ODN; and (iii) the genetic information can be completely removed using a simple organic solvent wash, and fully replaced with different genetic information during a short incubation step. This was done 5 times with no degradation of the surface throughout the subsequent immobilisations steps.

To demonstrate the specificity of the fluorous effect to immobilise ODNs into defined regions, without preventing the ability of the strands to participate in hybridisation events, we fabricated micro-patterned fluorous surfaces using standard optical lithography. These surfaces contained arrays of 50 × 50 μm squares modified with (heptadecafluoro-1,1,2,2-tetrahydrodecyl)trimethoxysilane, while the regions surrounding the squares were modified with *n*-decyltrichlorosilane (Fig. 1). A 1 μM solution of **F-DNA1** (a 16-mer ODN containing an 8 carbon fluorous tag, see ESI†) was added to the surface and incubated for 2 hours before washing using TE buffer (10 mM TRIS, 1 mM EDTA (pH 8.0)). A 1 μM solution of the fluorescently labelled (TAMRA) complementary sequence, **cDNA1**, was then added to the surface and incubated in a humidity chamber for 2 hours. The surfaces were then rinsed in turn with TE buffer and DI water, and imaged using fluorescence microscopy at 20× magnification (0.4 NA). As Fig. 1b demonstrates, the fluorous immobilisation technique leaves the **F-DNA1** strands available for hybridisation, and the **F-DNA1**/**cDNA1** duplex was confined to the patterned fluorous areas.

**Fig. 1:**
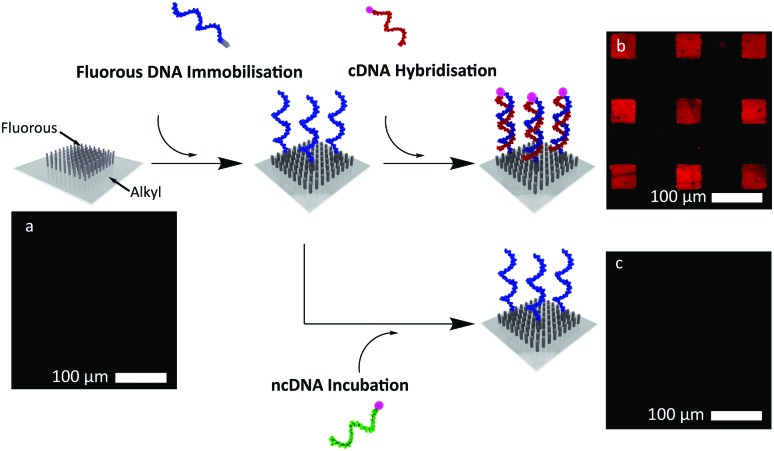
Schematic of fluorous immobilisation of the **F-DNA1**/**cDNA1** duplex on glass substrates in defined arrays. Schematic shows the immobilisation of **FDNA1** onto fluorous regions. The surface is then either incubated with the complementary strand (**cDNA1**) or a non-complementary strand (**ncDNA1**). Fluorescence images were obtained (a) before immobilisation; (b) after immobilisation of **cDNA1**; and (c) after incubation of **ncDNA1**.

One important issue in DNA microarray technology is non-specific binding, as this will ultimately limit both the sensitivity and the specificity of an assay.^6^ To test the extent of non-specific binding on our novel surfaces, we introduced a fluorescently labelled (TAMRA), non-complementary strand (**ncDNA1**) to a substrate modified with a fluorous-**F-DNA1** micro-pattern. No fluorescence was observed upon addition of the non-complementary sequence to the surface, Fig. 1c, demonstrating that **cDNA1** adhesion was mediated solely by hybridisation with the fluorous **F-DNA1**, see Fig. 1 and 2.

**Fig. 2:**
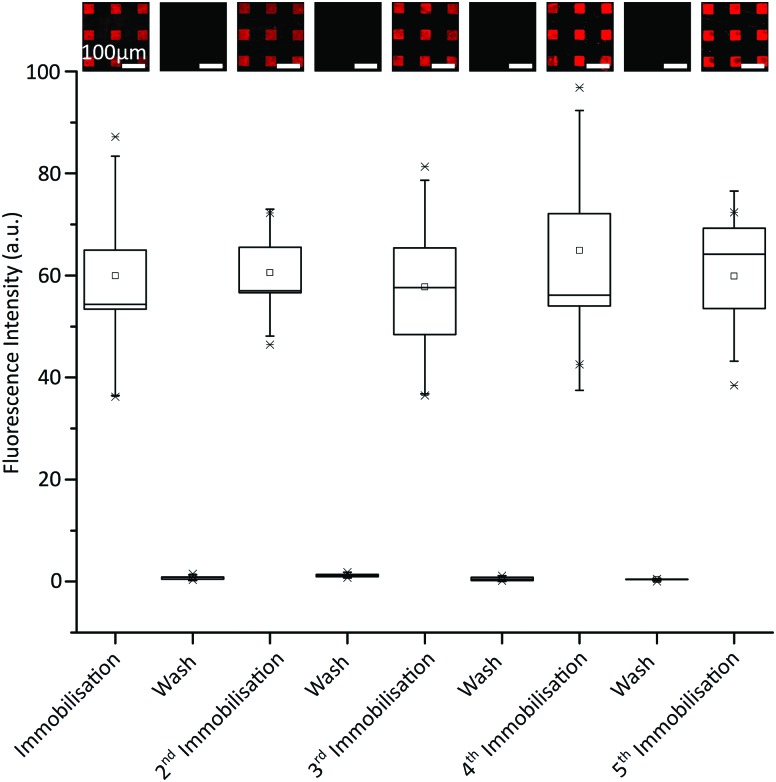
Following the immobilisation of **F-DNA1** and its hybridisation to **cDNA1**, the substrates were imaged using fluorescence microscopy. Fluorescence images were then taken following the complete removal of immobilised DNA after each washing step. This was repeated 5 times. Each image corresponds to the box plot beneath. The graph shows the change in fluorescence intensity of the different immobilisations and removals of the **F-DNA1**/**cDNA1** duplex from the surface.

We next investigated the reversibility of the immobilisation and the durability of the fluorous surface over five DNA removal-replacement cycles. A range of solvents were screened to determine the optimal washing protocol, which was found to be a solution of 50% v/v MeOH in phosphate-buffered saline (PBS) (1 M, pH 7.4) followed by a methanol rinse. No loss of fluorescence intensity was observed after 5 cycles of immobilisation and washing with MeOH/PBS (Fig. 2).

In order to assess if more complex information could be interchanged in place of less complex information on a surface, we synthesized a 32-mer ODN, **F-DNA2**, which contained a 5′fluorous tag and a complementary sequence, **cDNA2**, which contained an Alexa Fluor 488 tag. Further to this, we fabricated more intricate patterns using electron-beam lithography (Fig. 3), which contained feature sizes as small as 500 nm. Using our optimised conditions, described above, **F-DNA1** was immobilised, then hybridised with its complement **cDNA1** on the more intricate patterns. The duplex was then washed from the surface followed by the immobilisation of **F-DNA2** and hybridisation with **cDNA2**. Here we see the potential of this immobilisation chemistry for applications in re-writable DNA microarray technology, where the same surface can be used multiple times to detect different targets. Further to this, in the case of the **F-DNA2**/**cDNA2** duplex, the eight-carbon fluorous tag comprised less than 2% of the total mass. However, it was shown to be capable of immobilising the water-soluble ODNs with sufficient strength to remain bound to the surface through aqueous washes.

**Fig. 3:**
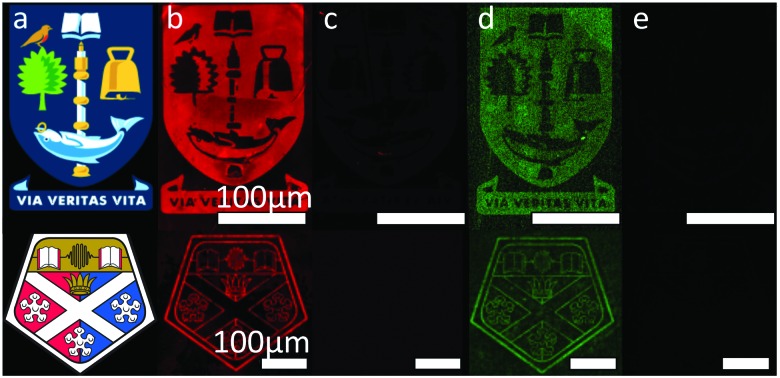
Fluorescence images taken following; (b) immobilisation of **F-DNA1**/**cDNA1** duplex; (c) complete removal of immobilised DNA by the washing step; (d) immobilisation of the **F-DNA2**/**cDNA2** duplex to the same pattern; (e) 2nd complete removal by washing. (c) and (e) are the combined images of the surface taken with filters for both TAMRA and Alexa Fluor 488. (a) Shows the original templates used for electron beam lithography.

To obtain further understanding of the nature of the fluorous immobilisation, we monitored the binding events using a Quartz-Crystal Microbalance (QCM) (Fig. 4). This enabled us to investigate the binding events as a reduction in the resonance frequency of an oscillating 5 MHz crystal due to an increase in mass.^28–30^ To determine the specificity of the interaction, immobilisation of **F-DNA1** was investigated using an unmodified silica QCM chip and a chip with a fluorous surface. Solutions of DNA (3.3 μM) were introduced to the chip using a flow cell at 40 μL min^–1^ and 20 °C. A decrease in frequency, representing an increase in surface mass, was observed upon addition of **F-DNA1** and adsorption was complete within 30 minutes for both surfaces.^31^ Although **F-DNA1** was adsorbed onto both fluorous and silica surfaces, after the introduction of the complementary sequence **cDNA1**, hybridisation was only observed in the case of **F-DNA1** immobilised onto the fluorous QCM chip. In the case of **F-DNA1** immobilised onto fluorous surfaces, the hydrophilic single-stranded DNA was directed away from the hydrophobic surface and toward the bulk solution, where it remained accessible to **cDNA1**. In the case where **F-DNA1** was immobilised on silica, it is possible that charge-inversion of the silanol surface by cations present in the buffer results in the single-stranded DNA lying flat against the surface, where it was inaccessible to hybridisation.

**Fig. 4:**
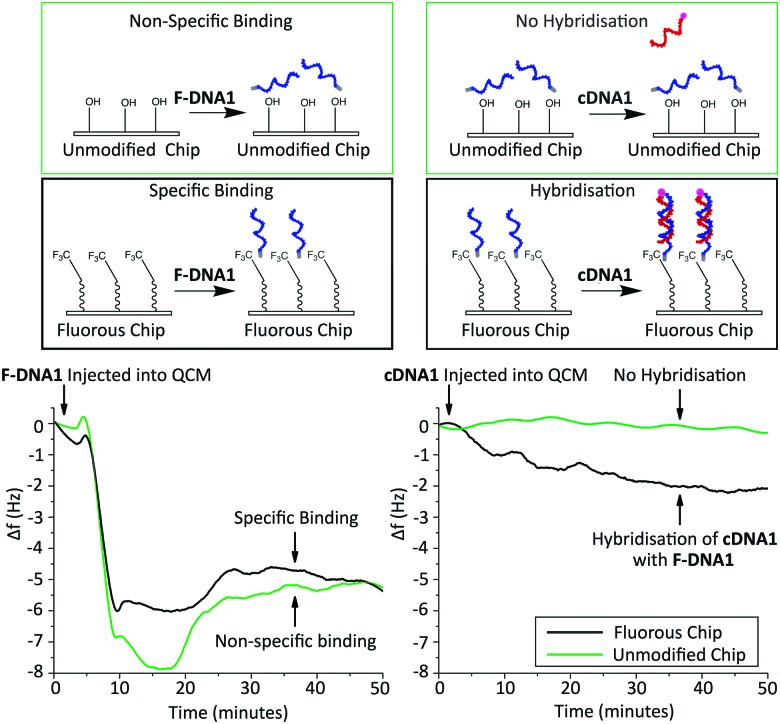
Schematics and real-time QCM measurement of DNA binding. Left: Comparison of **F-DNA1** binding to a fluorous modified surface (black) and a control surface with no fluorous modification (green). Right: Hybridisation of **cDNA1** to each surface after **F-DNA1** modification. A negative change in frequency (Δ*f*) represents an increase in mass on the surface.

In summary, we have demonstrated a novel method for the reversible immobilisation of DNA information onto surfaces. By developing lithographically patterned fluorous surfaces we remove the need to repeatedly direct the fluorous-modified biomolecules onto that surface for each subsequent “re-use”. The use of fluorous molecular tags enabled the immobilisation of ODNs to fluorous surfaces, and when used in conjunction with alkylated regions, allowed for the preparation of fluorescent patterns with low non-specific binding of the non-complementary strand to the sensing region and low non-specific binding of the target complementary strand to the non-sensing regions. ODNs immobilised using this method remained capable of hybridisation with complementary strands and displayed complete removal and replacement characteristics. This method enables the replacement of one DNA sequence for another, by a process that is repeatable and with no associated degradation of the efficiency of binding, with time. This reversible immobilisation chemistry could in the future be exploited across many research fields, particularly in DNA microarray development, where progress is already being made in fabricating re-usable sensing platforms.^32^

This work was supported by The EPSRC Centre for Doctoral Training (EP/F500424/1), the Royal Academy of Engineering (grant 10216/103), the EPSRC (grants EP/P51133X/1 and EP/N016874/1), and The Leverhulme Trust (grant RPG-2014-343). JC also acknowledges support from a personal EPSRC Fellowship (EP/K027611/1) and Biophononics ERC Advanced Investigator Award. The authors also wish to thank all the staff working in the James Watt Nanofabrication Centre for their support. All data relating to the work outlined in the article can be found here: http://dx.doi.org/10.5525/gla.researchdata.380.
