# Supplementary material for: Reversible DNA micro-patterning using the fluorous effect
Source: Chem Commun (Camb). 2017 Feb 23;53(21):3094–7. doi: 10.1039/c7cc00288b (PMC5358500; doi:10.1039/c7cc00288b)
Supplement: Supplementary file 1 [file CC-053-C7CC00288B-s001.pdf]

Electronic Supplementary Material (ESI) for ChemComm.
This journal is © The Royal Society of Chemistry 2017

Supporting Information: Reversible DNA Micro-patterning Using the Fluorous Effect

Experimental Section:

Materials. HPLC-purified DNA oligomers with several different modifications (Table 1) were purchased from Integrated DNA Technologies (IDT). Ultrapure water (18.2 MΩ.cm) was used for all solutions and protocols. All chemicals were obtained from Sigma unless otherwise stated. The oligomers were diluted to final concentrations in TE buffer and concentrations and purity was determined using UV-Vis spectrometry (Nanodrop 1000 Spectrophotometer, Thermo), prior to experiments.

Probe	Modification	Sequence
**FDNA1**	R^F HHH	TGC AGA TAG ATA GCA G
**FDNA2**	R^F HHH	CAT CAT GAA TTC CAT AAG CTT CAT GGA TCC AT
**cDNA1**	TAMRA	CTG CTA TCT ATC TGC A
**cDNA2**	Alexa Fluor 488	ATG GAT CCA TGA AGC TTA TGG AAT TCA TGA TG
**ncDNA1**	TAMRA	ATG ATG AAG CTT ATG ATG

Table 1: List of oligomers used, their base pair sequence and any modifications present.

Fluorous-tagged DNA Synthesis. Fluorous-tagged oligonucleotides were synthesised using standard solid-phase methods on an Applied Biosystems 392 DNA/RNA synthesiser. DNA synthesis reagents and solutions were purchased from Link Technologies Ltd. Oligonucleotides were purified by RP-HPLC, and characterised by MALDI-MS.

Oligonucleotide FDNA1: 5′ – R^F HHH TGC AGA TAG ATA GCA G – 3′ where R^F is a fluorous nucleotide and H is hexaethyleneglycol (HEG). Calculated m/z: 6528.22, observed m/z: 6507.59.
Oligonucleotide FDNA2: 5′ – R^F HHH CAT CAT GAA TTC CAT AAG CTT CAT GGA TCC AT – 3′ where R^F is a fluorous nucleotide and H is hexaethyleneglycol (HEG). Calculated m/z: 11300.01, observed m/z: 11314.14.

2-cyanoethyl (*1H,1H,2H,2H*-perfluorodecyl) diisopropylphosphoramidite

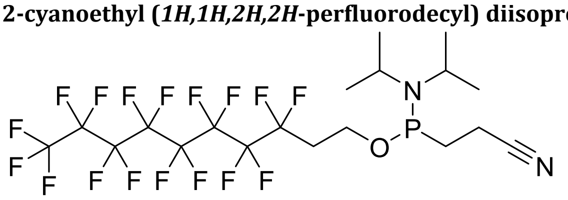

Under an inert atmosphere, *1H,1H,2H,2H*-perfluorodecanol (500 mg,0.77 mmol) and diisopropylethylamine (180 mg, 1.4 mmol) were dissolved in dry acetonitrile (6 mL). 2-Cyanoethyl N,N-diisopropylchlorophosphoramidite (357 mg, 1.5 mmol) was added. The reaction was monitored for completion by thin layer chromatography using ethyl acetate/hexane (1:3) as mobile phase (R_f of perfluorodecanol ~ 0.5, R_f of product ~ 0.7) and staining with acidified $KMnO_4$. Starting material was consumed after 2 hours. The reaction mixture was loaded directly onto a silica gel chromatography column and eluted with hexane/ethyl acetate/triethylamine (72:25:3), affording the product as a colourless oil (614 mg, 88 %). ^{1}H-NMR ($CDCl_3$, 500 MHz) δ: 1.20 (t, 12 H, J_{HH} = 7.3 Hz), 2.46 (m, 2 H), 2.64 (t, 2 H, J_{HH} = 6.4 Hz), 3.62 (m, 2 H), 3.78-4.02 (m, 4 H). ^{31}P NMR ($CDCl_3$, 200 MHz) δ: 148.6.

Micro-patterning of Substrates. Borate wafers (University wafers) were cleaned in toluene, acetone and IPA. They were then oxygen plasma ashered (2 minutes, 100 W) immediately before being transferred to a 1% v/v solution of n-decyltrichlorosilane (DTS) in toluene for 10 minutes. The surfaces were then cured at 100 °C for 30 minutes. Standard photolithography was used to micro-pattern the surfaces. Briefly, wafers were spin coated with S1818 (Shiply, USA) at 4000 rpm for 30 seconds to produce a ~1.8 µm thick layer. Micro-patterns were transferred onto the wafer by exposure to UV radiation (SUSS Microtec MA6, Germany) for 4.5 seconds through a chrome mask. The development of the S1818 resist was performed using a 1:1 ratio of Microposit developer (Shiply, USA) and RO for 1 minute. Samples were then dried under nitrogen. Following development, samples were oxygen plasma ashered for (2 minutes, 100 W) to remove the DTS silane from patterned areas. Samples were then sonicated in acetone for 5 minutes to remove the photoresist, rinsed with IPA and dried under nitrogen where they were then silanised, as described above, using (Heptadecafluoro-1, 1, 2, 2-tetrahydrodecyl) trimethoxysilane) (Gelest). To produce the crests, standard E-beam lithography was used, in place of photolithography, according to the procedure outlined in [1].

Immobilization & Hybridization of DNA. Immobilization of fluorous-tagged DNA (FDNA) on the surfaces was carried out in a simple humidity chamber. 1uM (unless otherwise stated) solutions of fluorous-tagged DNA were introduced into the chamber and incubated on FDTS micro-patterned surfaces at RT for 2 hours. Samples were rinsed with TE buffer and the complimentary DNA strand (1 uM unless otherwise stated) was introduced for 2 hours. Samples were then rinsed with DI water, TE buffer, dried under nitrogen then imaged. All experiments were carried out in triplicate.

Removal and Re-immobilization of DNA. Immobilized DNA was removed via the fluorous tag using a 1:1 solution of MeOH:PBS for 30 minutes under gentile agitation (Scheme 1). It was then rinsed with MeOH and dried under nitrogen. Re-immobilization was carried out using the same protocol as described above. Samples were protected from light to minimize photo-bleaching throughout the experiment.

QCM Apparatus. AT-cut quartz crystals with a fundamental resonance frequency of 5 MHz (25 mm) were obtained from Microvacuum Ltd (Hungry). These crystals were supplied with Au/SiO_2 coating that was used to graft silane molecules to using the same method outlined above. Crystals were mounted into an OWLS QCM-I (MicroVacuum, Hungary) attached to a mechanical pump to introduce the samples. DI water was flushed through until a stable baseline was established. The flow rate and temperature (40 µL/minute; 20 °C) remained constant throughout the experiment. Solutions were introduced slowly through an injection loop (total volume was 500 µL).

Fluorescence Microscopy. Surfaces were imaged using an Axio Observer Z1 (Carl Zeiss, Germany) under control of Axiovision software. Images were then analyzed using ImageJ software.

<u>Controls:</u>

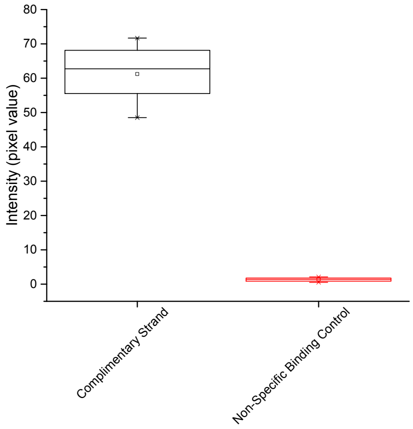

Figure 1: Fluorescence intensity observed from addition of non-complimentary sequence to fluorous micro-pattern.

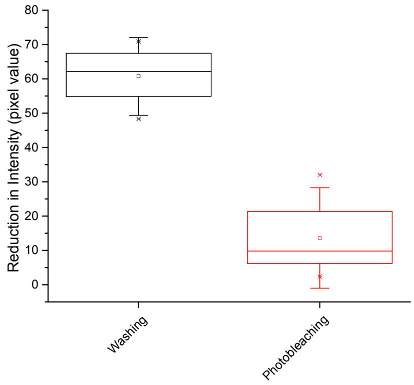

Figure 2: Reduction in fluorescence intensity due washing off fluorous tagged DNA using MeOH:PBS mixture and photobleaching.

Contact Angle Measurements:

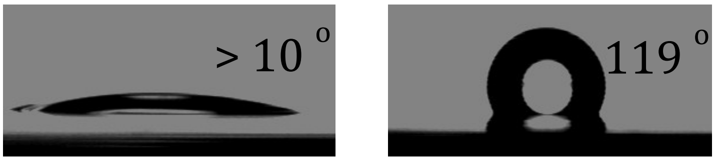

Figure 3: Contact angle measurements after cleaning surfaces, giving a contact angle of >10 °, and after silanising wafer with FDTS, giving a contact angle of 119°.

QCM Data:

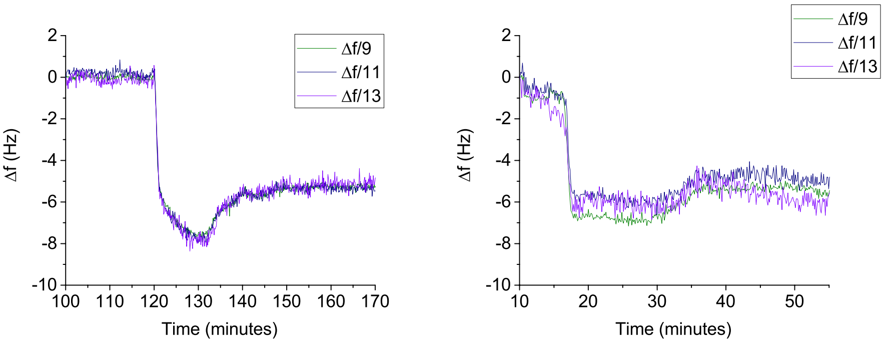

Figure 4: QCM data used in Figure 4 before smoothing. Plots show data from 3 overtones. (Left) F-DNA1 immobilisation onto unmodified QCM chip (Right) F-DNA1 immobilisation onto FDTS silanised QCM chip.

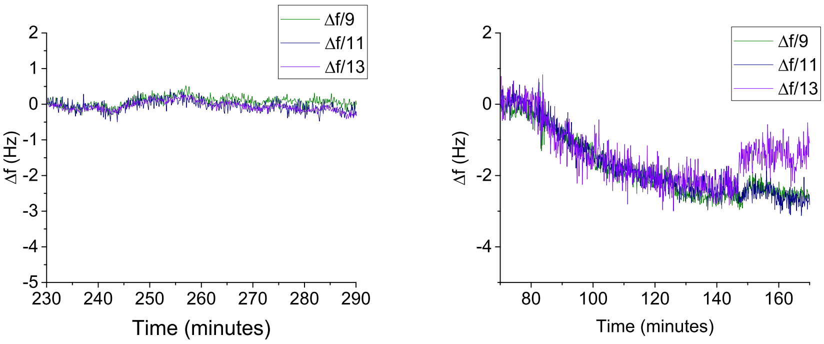

Figure 5: QCM data used in Figure 4 before smoothing. Plots show data from 3 overtones. (Left) cDNA1 injection into holder containing unmodified QCM chip (Right) cDNA1 injection into holder containing FDTS silanised QCM chip.

References:

1. Henry, S.L., et. *Organic & Biomolecular Chemistry*, 2016, **14**(4), 1359-1362.
